# Genetic characterization of *trh* positive *Vibrio* spp. isolated from Norway

**DOI:** 10.3389/fcimb.2013.00107

**Published:** 2013-12-25

**Authors:** Anette B. Ellingsen, Jaran S. Olsen, Per E. Granum, Liv M. Rørvik, Narjol González-Escalona

**Affiliations:** ^1^Department of Food Safety and Infection Biology, Norwegian School of Veterinary ScienceOslo, Norway; ^2^Norwegian Defence Research EstablishmentKjeller, Norway; ^3^Food and Drug Administration, Center for Food Safety and Applied NutritionCollege Park, MD, USA

**Keywords:** *tdh*, *trh*, *V. parahaemolyticus*, *V. alginolyticus*, MLST, urease, *Vibrio*, PCR

## Abstract

The thermostable direct hemolysin (TDH) and/or TDH-related hemolysin (TRH) genes are carried by most virulent *Vibrio parahaemolyticus* serovars. In Norway, *trh*+ *V. parahaemolyticus* constitute 4.4 and 4.5% of the total number of *V. parahaemolyticus* isolated from blue mussel (*Mytilus edulis*) and water, respectively. The *trh* gene is located in a region close to the gene cluster for urease production (*ure*). This region was characterized in *V. parahaemolyticus* strain TH3996 and it was found that a nickel transport operon (*nik*) was located between the first gene (*ureR*) and the rest of the *ure* cluster genes. The organization of the *trh-ureR-nik-ure* gene cluster in the Norwegian *trh*+ isolates was unknown. In this study, we explore the gene organization within the *trh-ureR-nik-ure* cluster for these isolates. PCR analyses revealed that the genes within the *trh-ureR-nik-ure* gene cluster of Norwegian *trh*+ isolates were organized in a similar fashion as reported previously for TH33996. Additionally, the phylogenetic relationship among these *trh*+ isolates was investigated using Multilocus Sequence Typing (MLST). Analysis by MLST or *ureR*-*trh* sequences generated two different phylogenetic trees for the same strains analyzed, suggesting that *ureR-trh* genes have been acquired at different times in Norwegian *V. parahaemolyticus* isolates. MLST results revealed that some pathogenic and non-pathogenic *V. parahaemolyticus* isolates in Norway appear to be highly genetically related.

## Introduction

*Vibrio parahaemolyticus* is a halophilic, Gram-negative proteobacterium ubiquitous in the marine and estuarine environment worldwide (Iida and Honda, 2006). It is one of the main bacterial pathogens associated with raw and undercooked seafood in Asia and the U.S. (Su and Liu, 2007). During the last decade, a *V. parahaemolyticus* clonal complex (mainly composed of O3:K6 serotype strains) has emerged, encompassing at least 14 serotypes (Okura et al., 2008), and described by the term “pandemic group” (Matsumoto et al., 2000; Okura et al., 2003; Nair et al., 2007).

Although the mechanism by which *V. parahaemolyticus* causes enteric disease is not fully understood, two virulence factors are usually associated with clinical isolates: the genes encoding for thermostable direct hemolysin (TDH), (*tdh*) and TDH-related hemolysin (*trh*) (Gonzalez-Escalona et al., 2006). Of the two, *trh* is less frequently observed than *tdh* (Nishibuchi and Kaper, 1995). Several variants of *tdh* have been identified, all of which are about 98% identical (Nishibuchi and Kaper, 1995). In the case of *trh*, only two gene variants have been described, denoted *trh1* and *trh2*; these are about 84% identical in sequence (Kishishita et al., 1992). The differences between *trh* and *tdh* are greater, showing only about 68% similarity (Nishibuchi et al., 1989). While *V. parahaemolyticus* can carry both genes and simultaneously express them, it appears that such isolates produces less TDH than *trh* negative isolates (Xu et al., 1994).

The frequency of *tdh* and/or *trh* expression among environmental *V. parahaemolyticus* isolates is typically <1%, but this may depend on location, sample source and detection method (Kaysner et al., 1990; Alam et al., 2002; Cook et al., 2002; Hervio-Heath et al., 2002; Martinez-Urtaza et al., 2008b). For example, between 49 and 78% of the sediment, water, or oyster samples from Willapa Bay (WA, U.S.) contained *trh*+ *V. parahaemolyticus* (Kaysner et al., 1990). In Norway, *trh*+ *V. parahaemolyticus* was isolated from only 4.4% of the *V. parahaemolyticus* positive blue mussel (*Mytilus edulis*) samples (Bauer et al., 2006) and 4.5% of water samples, and the *tdh* gene was not detected at all (Ellingsen et al., 2008).

Little is known about how these virulence factors are acquired by *V. parahaemolyticus*, although it has been suggested that the horizontal gene transfer of pathogenicity island(s) might be the acquisition mechanism (Nishibuchi and Kaper, 1995; Hurley et al., 2006; Izutsu et al., 2008). The *trh* gene is located in a region of ~16 kb that containing the *nik* (nickel acquisition system) and *ure* genes. This characteristic was observed in all *trh*+ strains analyzed (Park et al., 2000). A transposase gene has been identified next to the *trh* gene, suggesting the possibility that the entire region might have been transmitted into *V. parahaemolyticus* strains by an insertion sequence (IS)-mediated mechanism (Park et al., 2000). Isolates exhibiting *trh*+ are almost exclusively urease positive (*ure*+), which is not a typical characteristic for *V. parahaemolyticus* (Kaysner et al., 1994). This association was also observed in all Norwegian *trh*+ isolates (Bauer et al., 2006). Using long and accurate PCR (LA-PCR), Iida et al. (1998) found that the region between the *trh* and *ureC* genes was less than 8.5 kb. The gene organization of the *trh-ureR-nik-ure* gene cluster was determined in *V. parahaemolyticus* strain (TH3996) (Park et al., 2000), but whether other *trh*+ isolates have the same organization remains unknown.

Various typing methods are used to distinguish bacterial strains for epidemiological purposes (Foxman et al., 2005). However, conventional serotyping against O and K antigens appears to be of limited epidemiological value for *V. parahaemolyticus*, particularly given the recent emergence of O3:K6 and the other serotypes encompassed by the term “pandemic group” (Chowdhury et al., 2004a,b; Gonzalez-Escalona et al., 2008). Pulsed Field Gel Electrophoresis (PFGE) has been a favored method for genotyping *V. parahaemolyticus* isolates (Marshall et al., 1999) because of its high discrimination index, and has successfully been used in outbreak investigations worldwide. It has also been used to type *V. parahaemolyticus* isolates from seafood (Wong et al., 1999, 2000; Lu et al., 2000; McLaughlin et al., 2005). As PFGE is based on restriction enzyme digestion of total DNA, one potential drawback is that it may not be sensitive to recent genetic events such as horizontal gene transfer, which may be an important source of variability among isolates. An alternative approach, Multilocus Sequence Typing (MLST), is based on direct sequence analysis of housekeeping genes and a public database has recently been established to archive *V. parahaemolyticus* sequences (http://pubmlst.org/vparahaemolyticus) (Gonzalez-Escalona et al., 2008). MLST is comparable in cost to PFGE, and provides a different, if not better, details of the genetic relationships among isolates (e.g., evolutionary relationships) (Foxman et al., 2005). However, MLST analyses must be interpreted with caution since it is less sensitive for detecting recent genetic changes in populations that can be detected by PFGE, such as genome inversions, transposons, and plasmid, which in some cases will cause changes in their PFGE profiles.

The main objective of this study was to examine the genetic relationship among *trh*+ *V. parahaemolyticus* isolates from Norway by using a combination of MLST and *ureR-trh* region sequence analysis. Furthermore, the organization of the *trh-ureR-nik-ure* cluster was was explored in all of the *trh*+ isolates.

## Materials and methods

### Bacterial isolates

**Figure 1:**
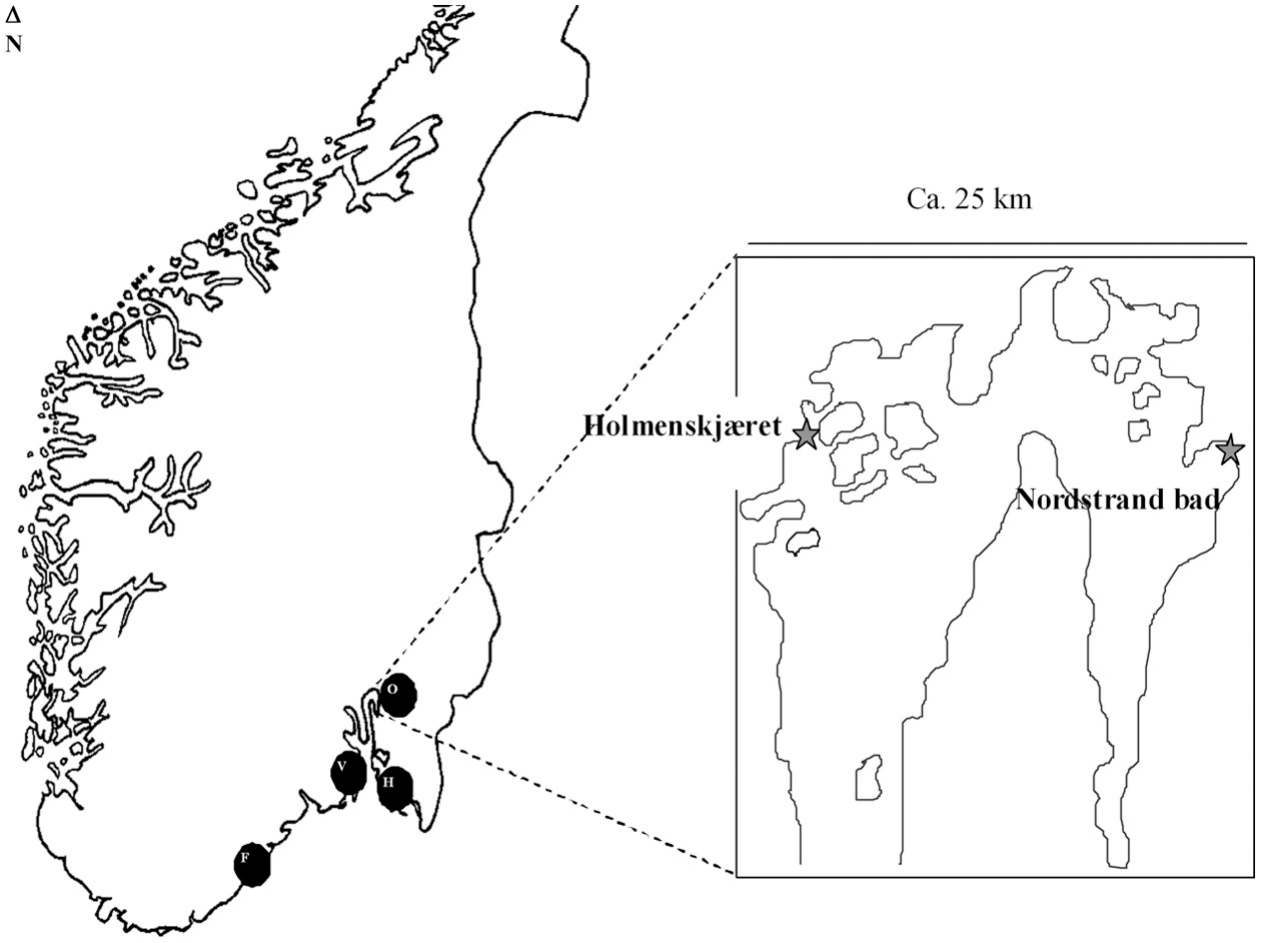
**Locations from which the Norwegian *trh*+ isolates originated (F, Flødevigen; V, Vallø; O, Oslofjord; H, Hvaler).** Locations within the Oslo fjord are indicated in the enlargement.

A total of 31 *V. parahaemolyticus* and two *V. alginolyticus* isolates were included in the present study (Table 1). Twenty-two of these strains were isolated from different regions in Norway (Figure 1) and nine comparator strains were from other regions of the world, including 4 from the United States which have been recently been characterized using MLST (Gonzalez-Escalona et al., 2008). Of the 22 Norwegian strains, 16 were *trh*+ (13 from environmental sources, 3 from clinical sources). The *trh*+ environmental strains were from water and blue mussel (*Mytilus edulis*) samples, collected from four different areas in Norway (Figure 1). The three *trh*+ clinical isolates were kindly donated by Jørgen Lassen from the Norwegian Institute of Public Health. The 6 *trh*– strains consisted of 4 environmental strains (3 from blue mussels, 1 from water) and two clinical strains. Norwegian *trh*– environmental isolates 551 and 580 were also included because they represent a larger PFGE cluster, consisting of *trh*–*V. parahaemolyticus* collected from several different locations along the Norwegian coast over several years; the PFGE pattern of these isolates appears very similar to the *V. parahaemolyticus trh*+ clinical isolate 224 (Ellingsen et al., 2008). Of the nine strains isolated in other countries, 5 were *trh*– and the other 4 were *trh*+. Both *V. alginolyticus* strains, 647va and 751va, were environmental isolates carrying a *trh*-like gene.

**Table 1:**
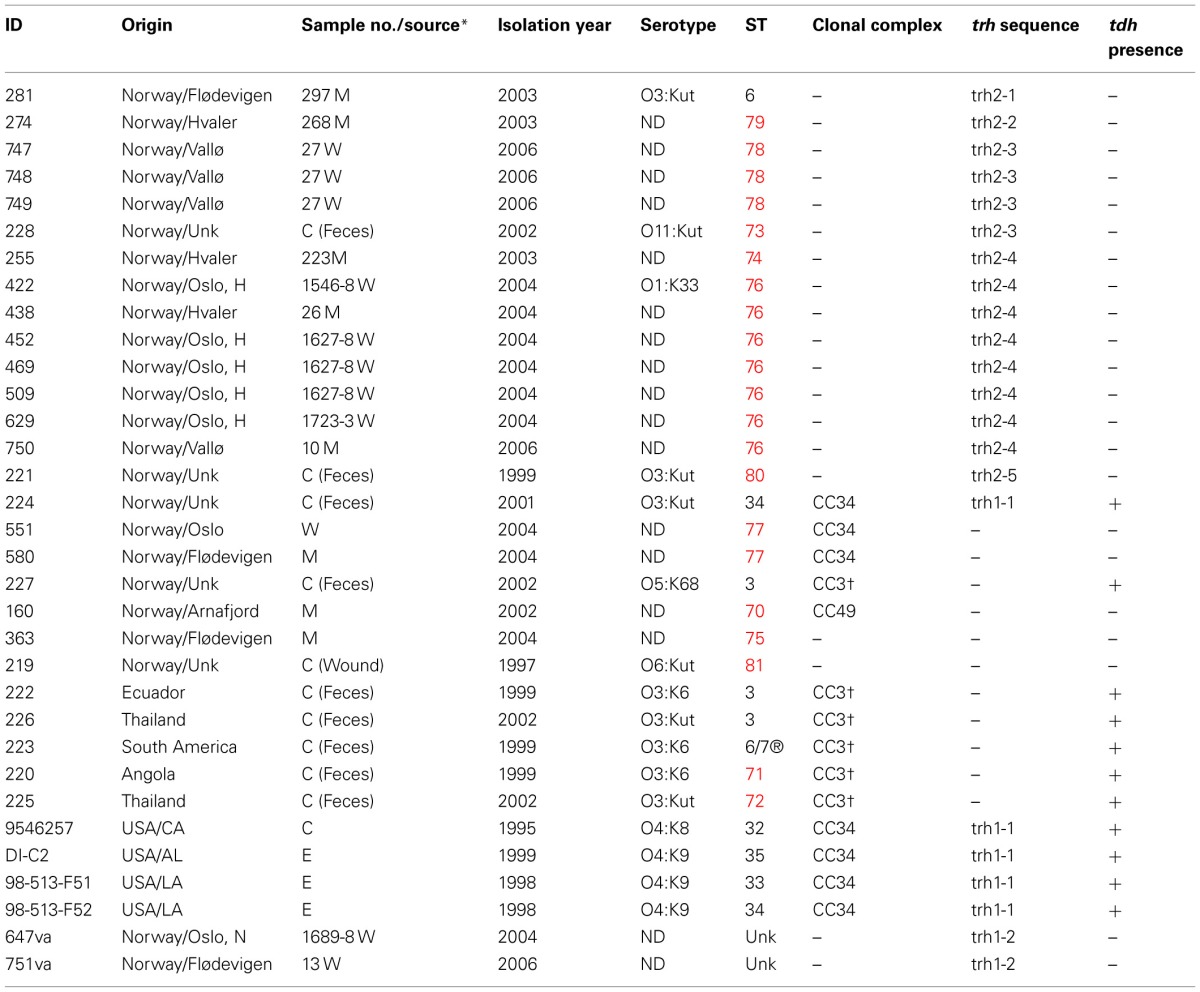
**Characteristics of the *Vibrio* spp. strains studied**.

^*^Sample type is indicated as follows; M, blue mussels (Mytilus edulis); W, water; C, clinical; E, environmental. In red are the new STs.

^†^Clonal complex with pandemic strains.

®The ST of isolate 223 were identical to ST-3 in 6/6 loci, but a complete recA sequence was not obtained due to technical difficulties (see text for more information).

ND, no determined; Unk, unknown.

All presumptive*V. parahaemolyticus* isolates were analyzed for the presence of oxidase, halophilic characteristics, (growth in presence of: 0, 3, 6, and 10% NaCl) and by the API-20E® Enteric Identification System (BioMerieux, France). The colony morphology was assessed on TCBS agar (Oxoid, Cambridge, UK). In addition, the identity of *V. parahaemolyticus* isolates were confirmed by *V. parahaemolyticus*-specific PCR targeting the *toxR* gene (Bauer and Rorvik, 2007).

### DNA preparation

DNA was extracted from one colony of cells suspended in 200 μ l sterile distilled water. The suspension was boiled for 10 min followed by centrifugation at 12,000 × g for 5 min. The supernatant (crude DNA) stored at −20°C until used as template in PCR.

### MLST

MLST was performed as described by Gonzalez-Escalona et al. (2008), using a set of PCR primers targeting *recA* (RecA protein), *dnaE* (DNA polymerase III, alpha subunit), *gyrB* (DNA gyrase, subunit B), *dtdS* (Threonine dehyrogenase), *pntA* (Transhydrogenase alpha subunit), *pyrC* (Dihydroorotase), *tnaA* (Tryptophanase). The PCR amplification and sequencing were performed as described at the *V. parahaemolyticus* MLST website (http://pubmlst.org/vparahaemolyticus).

### Amplification of the *trh-ureR-nik-ure* region

**Figure 2:**
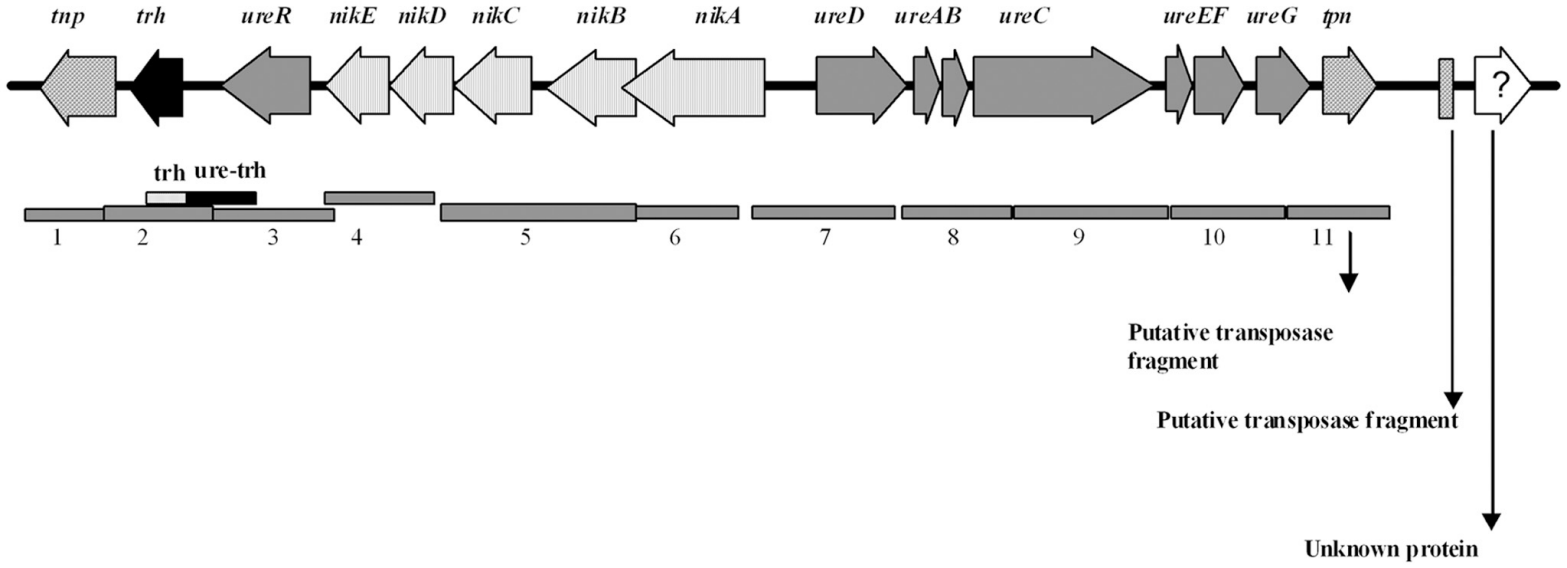
**Schematic representation of the *trh-ureR-nik-ure* gene cluster [adapted from Park et al. (2000)] together with an illustration of the gene products amplified by primer pairs 1–11 designed to assess the genetic organization (gray bars).** The fragment of the *trh* and *ureR-trh* primers also indicated.

A set of 12 primer pairs (Table 2) were designed using the Primer2 software (http://frodo.wi.mit.edu/cgi-bin/primer3/primer3_www.cgi) and the *trh*-*ureR*-*nik-ure* sequence AB038238.1 (Park et al., 2000) (antisense) as target. The target sequences of these primers were located in regions that spanned contiguous open reading frames (ORFs), as illustrated in Figure 2. Two additional sets of primers were used in order to amplify any partial *trh* (Honda et al., 1991; Honda and Iida, 1993)and *ureR* sequences from all *trh*+ isolates (Table 2). PCR was performed on all *trh*+ isolates, using the following conditions in a 50 μl reaction solution: 1× buffer (10 mM Tris-Cl pH 8.8, 1.5 mM MgCl_2_, 50 mM KCl, 0.1% Triton® X-100), 0.24 mM of each nucleotide (dNTP mix), 1.5 U μ*l*^−1^ Dynazyme II (all from Thermo Scientific, Vantoa, Finland), 20 pmol of the UtoxF primer, 20 pmol of the reverse primer, and 2 μl of sample DNA. The PCR running conditions were: an initial denaturation of 95°C for 4 min, 35 cycles of 94°C for 1 min, 50°C for 1 min and 72°C for 2 min, and a final elongation step of 72°C for 1.5 min in a Bio-Rad Ismart cycler (BioRad, Hercules, CA). Reference *V. parahaemolyticus* isolate AQ4037 (*trh1*+) was used as positive control. Reference *V. parahaemolyticus* isolate 222 (*trh*–) was used as negative control. All primers were synthesized by Medprobe (Oslo, Norway). All PCR amplicons resulting from *V. parahaemolyticus* isolate 438 were sequenced by Medprobe in order to confirm specific PCR amplification.

**Table 2:** **Primers employed to explore the organization of the *trh-nik-ure* region and to sequence the *ureR-trh* region in the *trh*+ isolates**.

**No.**	**Name**	**Primer sequence**	**DNA target (**5**′ **3**′)**	**Amplicon size (bp)**
1	Tnp-F	AATCAATCTCGTTGGGGTGA	34	927
	Tnp-R	ATTGATGAAGAGGCCATTCG	961	
2	Trh/ure-F	CGAATGGCCTCTTCATCAAT	941	1281
	Trh/ure-R	AAACATGGCCATCGAAAAAC	2222	
3	ureR1-F	GTTTTTCGATGGCCATGTTT	2222	1274
	ureR1-R	AGAGCCGCCAGCTAGGTATT	3476	
4	nikED	TTTGGCAAAGAGCTTGGAGT	3410	958
	nikED-R	GGTGGGGTTGAGTGAAGAGA	4368	
5	nikCB-F	GTCATCTGGCAGTGCTTTCA	4515	1893
	nikCB-R	CTCCTCGTGGTAACGGTGAT	6407	
6	nikA-F	ATCACCGTTACCACGAGGAG	6387	1392
	nikA-R	GGCGATCTCGCTATTTCTTG	7779	
7	ureD-F	CCCCGAGAGCAACAAATAAA	7883	1254
	ureD-R	TCTGTCTGATGACCGAGTGC	9137	
8	ureAB-F	GAATTTGGGCGACGTAAAGA	9215	1144
	ureAB-R	AGTGCCACCACCGATAAAAG	10358	
9	ureC-F	TGTTGGAAGCAGTCGATGAG	10420	1380
	ureC-R	GTTCCGCGAGGTAAAAACAA	11800	
10	ureEF-F	CCGGTGAAATTGCTCTTGTT	11839	1326
	ureEF-R	AAATTGTCGCCACCACTTTC	13164	
11	ureG-F	TAGCCCAGAGTTGGCAGATT	13174	985
	ureG-R	GGAAGAACTGCCTGAGAACG	14161	
trh^*^	Trh-L	TTGGCTTCGATATTTTCAGTATCT	1882	468
	Trh-R	CATAACAAACATATGCCCATTTCCG	1414	
trh-ureR	trurR2-F	AACGTAACTTTCAGATAATG	2889	1071
	trurR2-R	GTTCATCCGAACCTGGAGAA	1818	

All target sequences are reported with respect to AB038238.1 (Park et al., 2000).

* Primers designed by Honda et al. (1991), Honda and Iida (1993).

### trh and ureR sequence analysis

All partial *ureR-trh* and *trh* sequences genes were aligned and manually trimmed to be 1417 and 497 bp in length, respectively, using the Mega 3.1 software (Kumar et al., 2004), starting at position 1436 relative to AB038238.1 (GenBank, http://www.ncbi.nlm.nih.gov). Phylogenetic analysis of those sequences was performed using Mega 3.1 software (Kumar et al., 2004). Minimum evolution (ME) trees for both the *ureR-trh* sequences and partial *trh* sequences were constructed using the kimura-2 parameter model to estimate the genetic distances. Additional *trh* gene sequences available at GenBank database were included in the analysis (Figure 5). The statistical support of the nodes in the ME tree was assessed by 1000 bootstrap re-sampling. All amplified *trh* and *ureR* PCR products were sequenced by MedProbe (Oslo, Norway). The new sequences were deposited in GenBank under accession numbers FJ409538–FJ409547.

### tdh analysis

The presence of *tdh* was assessed by colony hybridization (DePaola et al., 2003) and/or by PCR amplification (Bej et al., 1999). The forward and reverse primers were 5′-gttctgatgagatattgtttgttg-′3 and 5′-gttggatatacacattaccaat-′3, respectively. The PCR reactions were performed as described above; except 0.2 mM of each nucleotide (dNTP mix) and 1.0 U μ*l*^−1^ Dynazyme II were used, with 30 cycles and an annealing temperature of 55°C.

## Results and discussion

The strains of *V. parahaemolyticus* and *V. alginolyticus* in this study presented the typical phenotypic characteristics of their type strains, ATCC 17802 and ATCC 17749, respectively. All *V. parahaemolyticus* strains resulted in positive PCR amplification of the *toxR* gene, but the two *V. alginolyticus* isolates (647va and 751va) did not produce any amplicons using the same PCR primers.

### MLST

The main objective of the present study was to establish the genetic relationships among *trh*+ *V. parahaemolyticus* isolated from Norwegian environmental (mussels and seawater) or clinical sources.

**Figure 3:**
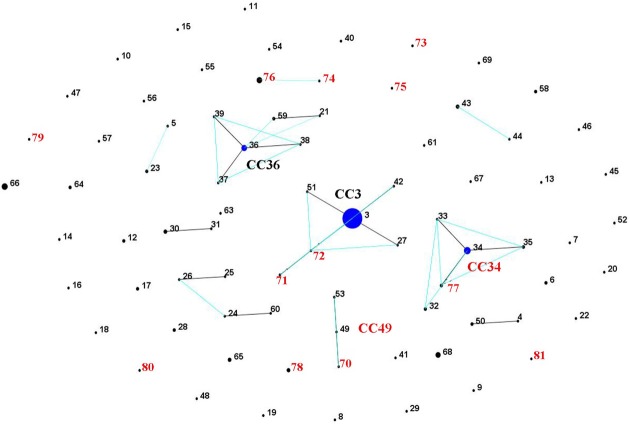
***V. parahaemolyticus* population “snap-shot” based on the novel STs described in the present study and previously described STs submitted to the MLST database (Gonzalez-Escalona et al., 2008), using eBURST v3 (http://eburst.mlst.net).** Four different clusters were observed using stringent criteria (5/7 shared alleles). Isolates from the present study are indicated by red numbers. The clonal complexes CC are indicated (the new one in red CC49), and the predicted ancestral clones are indicated by blue dots. Single locus variants (SLV) are indicated by dark lines, while double locus variants (DLV) are turquoise. The number of isolates in each ST is indicated by different dot sizes.

The 22 Norwegian *V. parahaemolyticus* isolates were differentiated into 15 different sequence types (STs), of which 12 had not previously been reported in the *V. parahaemolyticus* MLST database (Table 1, Figure 3), indicating a high degree of genotypic diversity among these isolates. This confirms earlier reports of diversity among *V. parahaemolyticus* found in other geographic locations (Gonzalez-Escalona et al., 2008; Johnson et al., 2009; Rodriguez-Castro et al., 2009). Three of the STs we identified in the Norwegian samples had already been described in the MLST database: ST3, ST6, and ST34. Interestingly, ST3 is believed to be the ancestral type of pandemic CC (Gonzalez-Escalona et al., 2008), ST6 is a singleton that does not appear related to any known CC, and ST34 is believed to be the ancestral type of CC34 (Gonzalez-Escalona et al., 2008). Our analysis also revealed the existence of a new *V. parahaemolyticus* CC, CC49 (Figure 3, Table 1).

Of the nine non-Norway isolates*V. parahaemolyticus* used as reference strains, four (9546257;DI-C2, 98-513-F51 and 98-513-F52) had been previously analyzed by MLST and belonged to a clonal complex (CC34) (Gonzalez-Escalona et al., 2008) and the other five were ST3, except strain 223. It was difficult to obtain good quality *recA* sequences for most isolates studied, specifically at both ends of the sequences. In the case of clinical *V. parahaemolyticus* isolate 223; a complete *recA* sequence was not obtained. However, the six remaining loci were identical to those of isolates belonging to CC3 (ST3). This suggests that isolate 223 belongs to CC3 and is a single sequence variant (SLV) on *recA* locus of its ancestral type (ST3). The majority of these CC3 isolates were acquired in Asian or South American countries (Table 1), further emphasizing the worldwide distribution of the pandemic clonal group.

Only 16 of the 22 Norwegian *V. parahaemolyticus* isolates were carrying the *trh* gene. These isolates were subdivided into eight different STs. Interestingly, seven of these *trh+ V. parahaemolyticus* environmental isolates were shown to be identical by MLST (ST-76), even though they originated from different sources, locations and collection years (2004 and 2006). This indicates their persistence in the coastal environs of Norway. One *trh*+ environmental isolate (281) displayed the same ST (ST6) as an environmental *V. parahaemolyticus* isolate from Chile (Gonzalez-Escalona et al., 2008). However, the Chilean isolate (PMA 112) was negative for both virulence factors. Further analyses using whole genome sequencing could provide additional evidence about how similar or related these two isolates are.

In contrast to the similarity seen among some environmental strains, each of the three Norwegian *trh*+ clinical isolates belonged to different STs, two of which were novel (ST-73 and ST-80). The third *trh*+ clinical isolate (224) belonged to ST-34. This ST was previously observed for an O4:K9 isolate collected from environmental sources in Louisiana in 1998. Intriguingly, ST-34 had been suggested to be the ancestral clone of CC34, which consists of non-pandemic isolates (Gonzalez-Escalona et al., 2008). Isolate 224 may further indicate an association of this cluster with human disease (Gonzalez-Escalona et al., 2008), as well as strengthen the possible role of ST-34 as the ancestral clone of this clonal complex. Clinical isolate 224 (*tdh*+/*trh*+) is a single locus variant from two environmental isolates 551 and 580 (*tdh*–/*trh*–) belonging to ST-77. The exact origin of 224 is unknown: the patient did not report any stay abroad prior to the infection; however, it is possible the patient ate food that had been re-contaminated during preparation or the patient had consumed imported seafood.

Finding two identical STs in different regions of the world provides evidence that a clonal complex, different from the pandemic clonal complex, is spreading outside its original geographic location. There could be numerous routes for such rapid worldwide dissemination, for transport of contaminated ballast water (McCarthy et al., 1992; McCarthy and Khambaty, 1994), imported food, the motion of oceanic currents, (Martinez-Urtaza et al., 2008a) or human travel activity (Lantagne et al., 2013).

One isolate (novel ST-81) was identified to be *trh–/tdh–*, and recovered from a wound infection (Table 1). One isolate 227 was ST-3 (“pandemic” clone) and isolated from a clinical case in Norway (Table 1). However, it is unclear whether the pandemic clone has reached the Norwegian environment since there were no epidemiological links to a Norwegian source. Pandemic *V. parahaemolyticus* had been previously reported in the European environment (Martinez-Urtaza et al., 2005; Quilici et al., 2005; Caburlotto et al., 2008).

MLST was also performed on the two *V. alginolyticus* sequences using the same conditions as for *V. parahaemolyticus*. However, the PCR did not result in amplification for five of seven MLST loci. Sequences obtained from *gyrB* and *dtdS genes* were not identified in the MLST database (http://pubmlst.org/vparahaemolyticus).

### Amplification of the trh-ureR-nik-ure region

**Figure 4:**
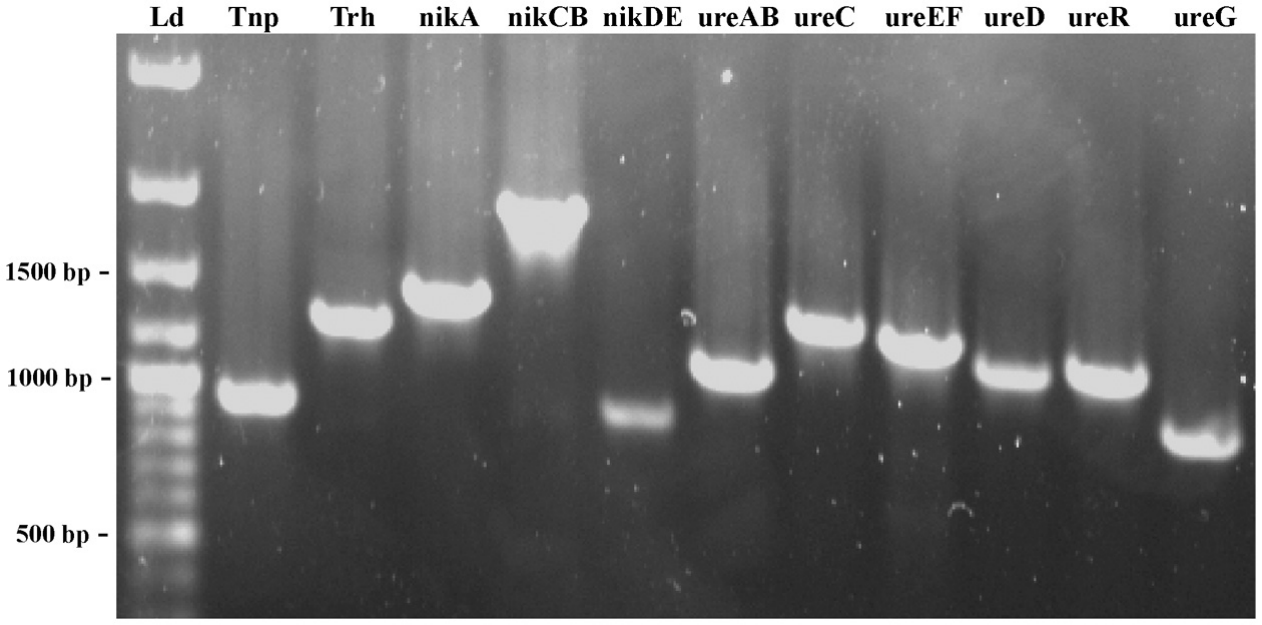
**PCR products amplified by each of the primers set designed to determine the organization of the *trh-ureR-nik-ure* region in each *trh*+ isolate.** The fragments of 500, 1000 and 1500 bp are indicated. The name of each gene target is indicated at the top of each lane. Ld—GeneRuler™ 100 bp Plus DNA Ladder (Fermentas).

The organization of the *trh*-*ureR*-*nik-ure* region previously described in most *trh*+ isolates (Park et al., 2000) was also explored in Norwegian *trh*+ *V. parahaemolyticus* and *V. alginolyticus* isolates. Amplification of the *trh-nik-ure* region of these isolates resulted in PCR products of correct length, but with exceptions for six isolates (Figure 4, Table 2). Isolate *V. parahaemolyticus* 647 Va did not produce any amplicon with primer pair 1 (*tnp*). Isolates 221, 228, 747, 748, and 749 did not produce any amplicon with primer pair 2 (*trh*) (Figures 2, 4). Additionally, weak amplicons of the correct size were observed with primer pair 2 and 3 (*trh* and *ureR*) for all isolates, except for isolates 224, 647Va, and 751Va for which no amplicons were obtained. Comparing these sequences to those previously submitted to NCBI revealed that the targeted regions of the forward and/or reverse primer of these primer sets (2 and 3) were variable between *trh1* and *trh2*, thus explaining the observation of weak and secondary products of different sizes for the *trh2* sequences (data not shown). In conclusion, these primer sets were inadequate for isolates harboring the *trh2* gene. We avoided the challenge presented by these two primer pairs by using two additional primer sets (trh and trh-ureR (Honda et al., 1991; Honda and Iida, 1993).

Sequences obtained from isolate 438 confirmed that the PCR products were the intended targets (data not shown). The gene organization of the *trh*-*ureR*-*nik-ure* region of all *trh*+ isolates was shown to be identical to the cluster described by Park et al. (2000). One of the *V. alginolyticus* isolates (647) was negative for primer pair 1 (*tnp*). Whether this result is due to the absence of this gene, or if there are mutations in the target sequence(s) is currently unknown. Sequencing the genome of these strains will reveal in which genomic region the *trh* gene is present, identify the surrounding genes, and also provide a possible explanation for the presence of *trh* in these isolates.

### Partial trh and ureR sequences

**Figure 5:**
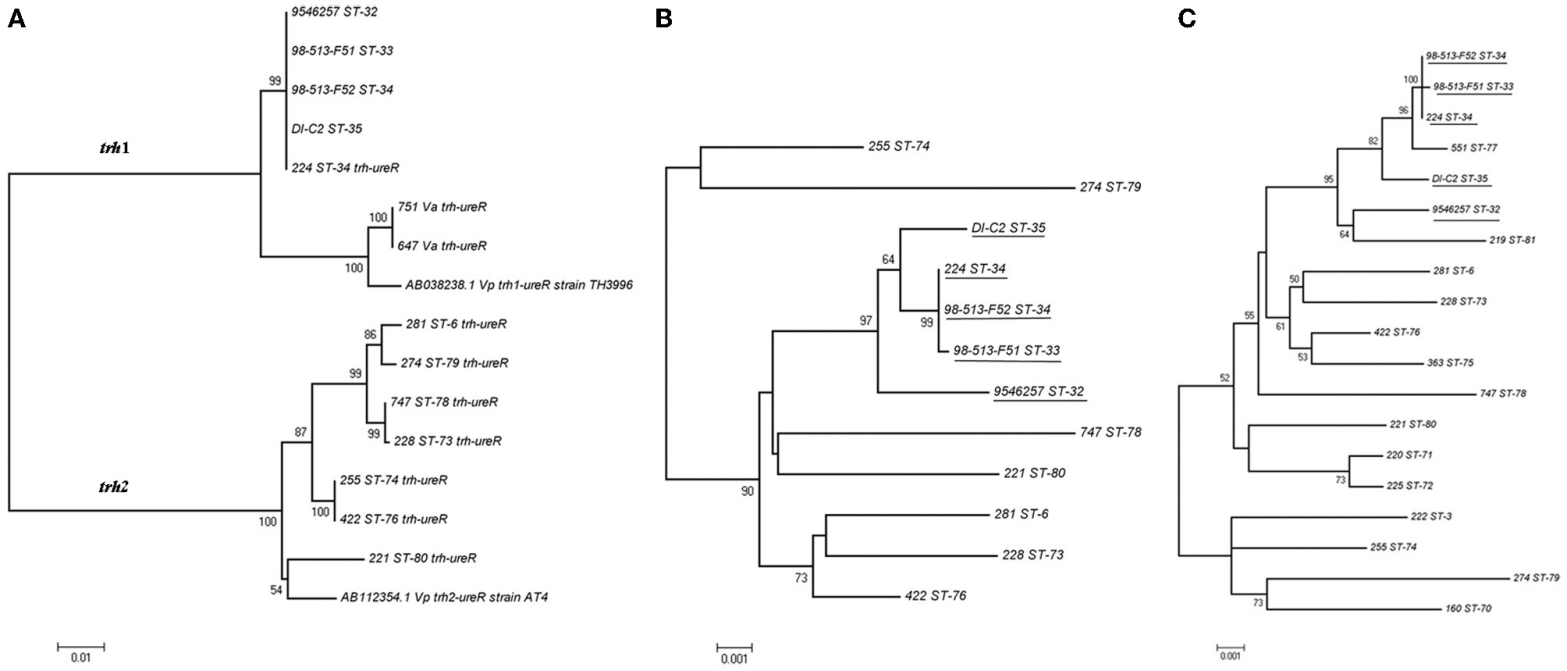
**Minimum evolution trees (ME) trees constructed from either the concatenated sequences of the seven loci (MLST) or *ureR-trh* region of each of the sequence types (STs) obtained in this study. A)** ME tree of the *ureR-trh* region obtained for the *trh*+ isolates together with representative sequences of either *trh*1 or *trh*2 retrieved from GenBank. **(B)** ME tree of the concatenated sequences of the seven loci of the *trh*+ isolates. **(C)** ME tree of concatenated sequences of all the *V. parahaemolyticus* isolates from this study. Underlined are the isolates carrying *ureR-trh1* region. Scale represents the evolutionary distance and boot-strap values over 50% are shown in the branches.*ureR-trh* region sequences of *V. alginolyticus* isolates are indicated in the figure as Va.

Sequence analysis performed on *ureR-trh* region sequences (1417 bp) obtained from the 22 *trh*+ isolates (Table 1) showed a total of eight different sequences (Figure 5A). In contrast, an analysis of only the partial *trh* sequences resulted in seven different sequences (Table 1). This discrepancy was a result of a single nucleotide difference in the *ureR*-*trh* intergenic region (position 698) between isolates 747 (ST-78) and 228 (ST-73). Alignment of the partial *trh* gene sequences of all *trh*+ isolates with representative *trh*1 and *trh*2 sequences retrieved from GenBank revealed that *V. parahaemolyticus* isolates 224 (ST-34), 9546257 (ST-32), DI-C2 (ST-35), 98-513-F51 (ST-33), and 98-513-F52 (ST-34) and both *V. alginolyticus* isolates harbored the *trh1* sequence type, while the remaining isolates carried *trh2* sequence type (Figure 5A). The partial *trh* sequences of the two *V. alginolyticus* isolates (647Va and 751Va) were identical. The three *trh1* sequences had a four nucleotide insert (GATA) 160 bp upstream of the *trh* start codon (position 2090, relative to AB038238.1), and a deletion 101 bp upstream. These mutations were in the non-coding region. The *trh1* gene is occasionally associated with the presence of *tdh* (as observed in isolate 224), although the two *V. alginolyticus* isolates in this study were negative for this gene by PCR. The *trh2* type had previously been described in a *V. alginolyticus* isolated from Alaskan oysters (Gonzalez-Escalona et al., 2006). This is the first report of *trh1* in *V. alginolyticus*. The presence of *trh* in other *Vibrio* spp. than *V. parahaemolyticus* has previously been described, and suggests that environmental *Vibrio* spp. may be the reservoir for the *trh* gene (Gonzalez-Escalona et al., 2006; Masini et al., 2007). This is not unique, since another *V. parahaemolyticus* pathogenicity determinant (*tdh* gene) was previously identified in a *V. alginolyticus* isolate (Cai et al., 2007). This re-enforced the reported highly mobile nature of these genes in marine environment among *Vibrio*.

Comparison of the minimum evolution (ME) trees generated from the concatenated sequences of the seven genes (MLST) of all *trh*+ *V. parahaemolyticus* isolates and their 1417 bp *ureR*-*trh* region, revealed different evolutionary stories between the housekeeping genes and this virulence region (Figures 5A,B). As expected, the *ureR*-*trh1* region was identical among the members of the clonal complex 34 (CC34), which all belong to closely related STs (Figure 5B). In the case of the *V. alginolyticus* isolates, this region was more similar (99%) to the one in *V. parahaemolyticus* (TH3996) than to the one present in CC34 isolates (96%), indicating that this region of *V. alginolyticus* was acquired from a different genetic background that the one in CC34 isolates. On the other hand, even though strains 225 and 422 contained identical *ureR-trh*2 region, they belonged to very distant STs, (ST-74 and ST-76, respectively), both located at distant part of the ME MLST tree.

These results also demonstrate how MLST, in conjunction with *ureR-trh* cluster analysis, is a powerful technique for revealing evolutionary events inside the *V. parahaemolyticus* species. It has been suggested that *Vibrio* spp. are more vulnerable to horizontal gene transfer than the other prokaryotic species (Dryselius et al., 2007), and that frequent recombination events further drive clonal diversification (Gonzalez-Escalona et al., 2008). In this study, the conservation of the *ureR*-*trh*1 among isolates belonging to CC34 (Gonzalez-Escalona et al., 2008) suggests that this region was acquired before those isolates started to diverge.

MLST analyses indicate that *V. parahaemolyticus* isolates 225 (ST-74) and 422 (ST-76), both containing an identical *ureR*-*trh*2 region, have diverged in the past (Figure 5B), and suggest that this region was acquired recently. Another possible explanation could be that this region evolved or changed more slowly in comparison with the other housekeeping genes (MLST). However, since MLST analyzes housekeeping genes, presuming a recent acquisition of that region seems a more parsimonious explanation. Whether this *ureR*-*trh*2 region was acquired recently by only one or both of these isolates, remains unknown.

Furthermore, analysis of the ME tree generated with the concatenated sequences of the seven genes of all the *V. parahaemolyticus* isolates in this study, including *tdh+/trh–* and *tdh–/trh–* isolates, showed that some isolates were highly related despite their *tdh* or *trh* content (Figure 5C). These findings support previous reports that these elements could be laterally acquired (Nishibuchi and Kaper, 1995; Park et al., 2000). An example is strain 551 (ST-77), which, although not carrying either determinant, is nonetheless closely related to isolates belonging to the ancestral type of CC34 (ST-34) which contains *ureR-trh*1 region (Figure 5C). Isolate 551 is a SLV of isolates belonging to ST-34 and differed by six nucleotide changes on locus *pyrC*. This amount of changes in this locus, compared to the other six, indicates that these different variants of the locus have arisen by recombination rather than mutation (Gonzalez-Escalona et al., 2008). The lack of *ureR-trh*1 region in this isolate (551) could be due to a loss of this region or that this isolate did not acquired it. Whole genome sequencing of these strains will provide some insights into the loss/acquisition of the *ureR-trh*1 region by these isolates.

### Geographical distribution of trh+ *V. parahaemolyticus* STs in norway

The isolation of multiple *trh*+ *V. parahaemolyticus* strains from all four sampling sites shows that these types of isolates are prevalent on the coast of Norway (Figure 1, Table 1). MLST analysis showed that most areas have unique *V. parahaemolyticus* populations, as evidenced by their different STs; exceptions were ST-76 and ST-77, which were isolated from 3 (Hvaler, Vallø, and Oslo) and 2 (Oslo and Flødevigen) of the four areas tested, respectively. The presence of the same ST across those different areas has no easy explanation although this could be due to either ocean currents or interchange of blue mussels among different Norwegian coastal areas.

One area of special interest was Hvaler, where *V. parahaemolyticus* strains belonged to three different ST with different degrees of similitude. For example ST-79 (*trh2-2*) and ST-74 (*trh2-4*) were located closely in the concatenated sequence ME tree (Figure 5B), and carried different *trh* gene variant. While strain 438 ST-76 (*trh2-4*), was identical to other strains isolated from Oslo and Vallø, it also carried the same *trh* gene variant than ST-74. This detail indicates that there are different populations of *trh*+ *V. parahaemolyticus* in the same area (Hvaler), carrying the same *trh* variant, suggesting horizontal gene transfer of this gene among them. Interestingly, the ST-76 strains in Hvaler and Vallø were isolated from blue mussels while the ST-76 strains from Oslo were all isolated from water.

Also, it appears to be a high prevalence of *trh2* vs. *trh1* gene in environmental *V. parahaemolyticus* in Norway, but in order to offer a more conclusive answer to this phenomenon more strains from different areas, seasons, and years, should be analyzed. *trh2+* was observed in all environmental *trh*+ *V. parahaemolyticus* and two of the 3 Norwegian clinical cases. In the other hand, *trh1* gene was observed exclusively in the *V. alginolyticus* analyzed, and in one of the 3 Norwegian clinical cases. *trh1+* *V. alginolyticus* were isolated in two distant regions Flødevigen and Oslo, Nordstrand bad, indicating that there appears to be strains in those regions that act as a reservoir of *trh* genes in the Norwegian aquatic environment.

## Conclusion

Our study presents new information regarding the genetic relationship among *trh*+ isolates, and introduces a PCR screening method demonstrating that the organization of the *trh-ureR-nik-ure* gene cluster found in the Norwegian *trh*+ *V. parahaemolyticus* isolates is identical to the one previously described for strain TH3996 (Park et al., 2000). Overall, these results showed that the genetic background of *trh+ V. parahaemolyticus* in Norwegian coasts is highly complex and there appears to be a movement of the same, or highly similar, strains between coastal regions. Additionally, the current study is (to the author's knowledge) the first to indicate a genetic relationship between *trh*+ and/or *tdh+* *V. parahaemolyticus* and non-pathogenic environmental isolates. These intriguing results warrant closer examination of the *trh*+ and/or *tdh*+ isolates [224 (ST-34) and 228 (ST-73)] and their non-pathogenic “relatives” [551 (ST-77) and 281 (ST-6)], which may provide unique information regarding the transfer of genetic elements carrying *trh* and/or *tdh.* In conclusion, MLST and *ureR-trh* sequence analysis generated two different evolutionary trees, suggesting that *ureR-trh* genes have been acquired or lost at different times by Norwegian *V. parahaemolyticus* isolates.

## Author contributions

Anette B. Ellingsen, Jaran S. Olsen, Per E. Granum, and Liv M. Rørvik designed parts of the study. Anette B. Ellingsen and Liv M. Rørvik organized the collection of Vibrios. Anette B. Ellingsen, Jaran S. Olsen, and Narjol González-Escalona performed laboratory and phylogentic analyses. Anette B. Ellingsen, Liv M. Rørvik and Narjol González-Escalona analyzed the data obtained from the whole study. Anette B. Ellingsen, Jaran S. Olsen, Per E. Granum, Liv M. Rørvik and Narjol González-Escalona wrote the final manuscript. All authors read and approved the final manuscript.

### Conflict of interest statement

The authors declare that the research was conducted in the absence of any commercial or financial relationships that could be construed as a potential conflict of interest.
